# Supplementary material for: A widespread gut bacterial lineage distinguished by redox metabolism and phage defense
Source: bioRxiv. 2026 Apr 1:2026.03.31.715625. Preprint. [Version 1] doi: 10.64898/2026.03.31.715625 (PMC13060105; doi:10.64898/2026.03.31.715625)
Supplement: 2 [file NIHPP2026.03.31.715625v1-supplement-2.pdf]

bioRxiv preprint doi: https://doi.org/10.64898/2026.03.31.715625; this version posted April 1, 2026. The copyright holder for this preprint (which was not certified by peer review) is the author/funder, who has granted bioRxiv a license to display the preprint in perpetuity. It is made available under aCC-BY 4.0 International license.

SUPPLEMENTAL FIGURES AND FIGURE LEGENDS

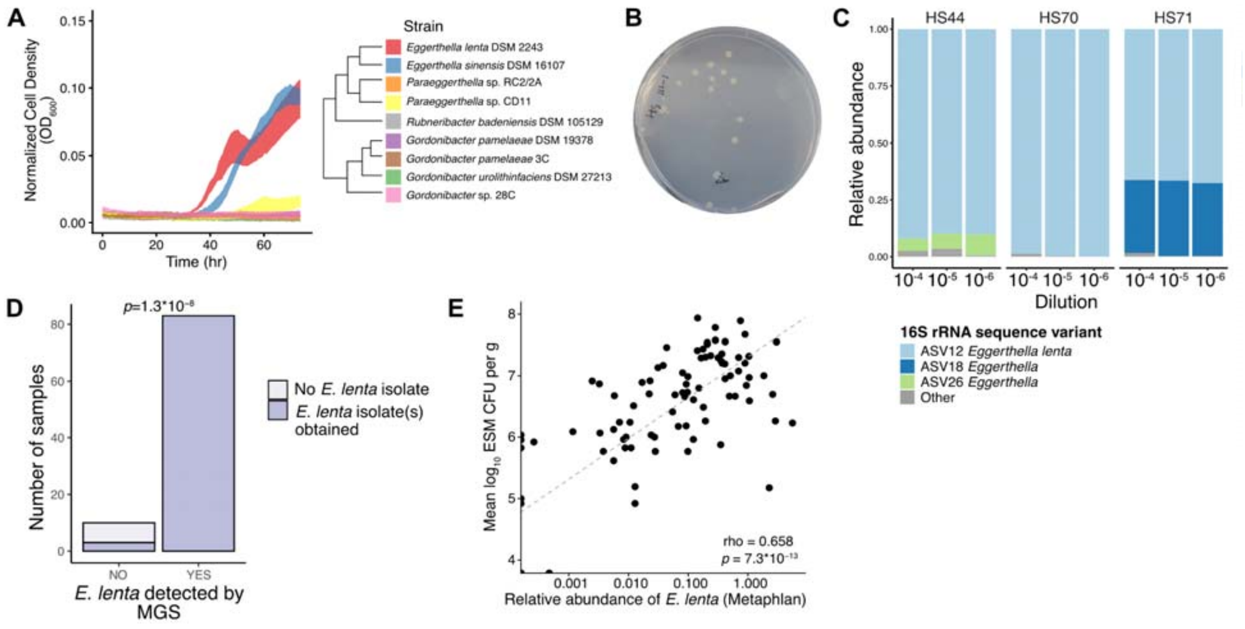

Figure S1. Selective isolation of *E. lenta* from human stool samples. (A) ESM supports the anaerobic growth of *E. lenta* and not closely-related members of the *Eggerthellaceae* family (phylogenetic relationships shown from GTDB). **(B)** Example ESM agar plate with *E. lenta* colonies. (**C)** 16S rRNA gene sequencing-based validation of the selectivity of ESM for amplicon sequence variants (ASVs) assigned to genus *Eggerthella* (n=3 stool samples). **(D,E)** Culture-dependent (ESM) and -independent (MetaPhlAn analysis of metagenomic data) detection of *E. lenta* in the ImmunoMicrobiome cohort[31] show consistent presence/absence **(D)** and abundance **(E)**. Statistics: **(D)** Fisher's exact test; **(E)** Spearman correlation.

bioRxiv preprint doi: https://doi.org/10.64898/2026.03.31.715625; this version posted April 1, 2026. The copyright holder for this preprint (which was not certified by peer review) is the author/funder, who has granted bioRxiv a license to display the preprint in perpetuity. It is made available under aCC-BY 4.0 International license.

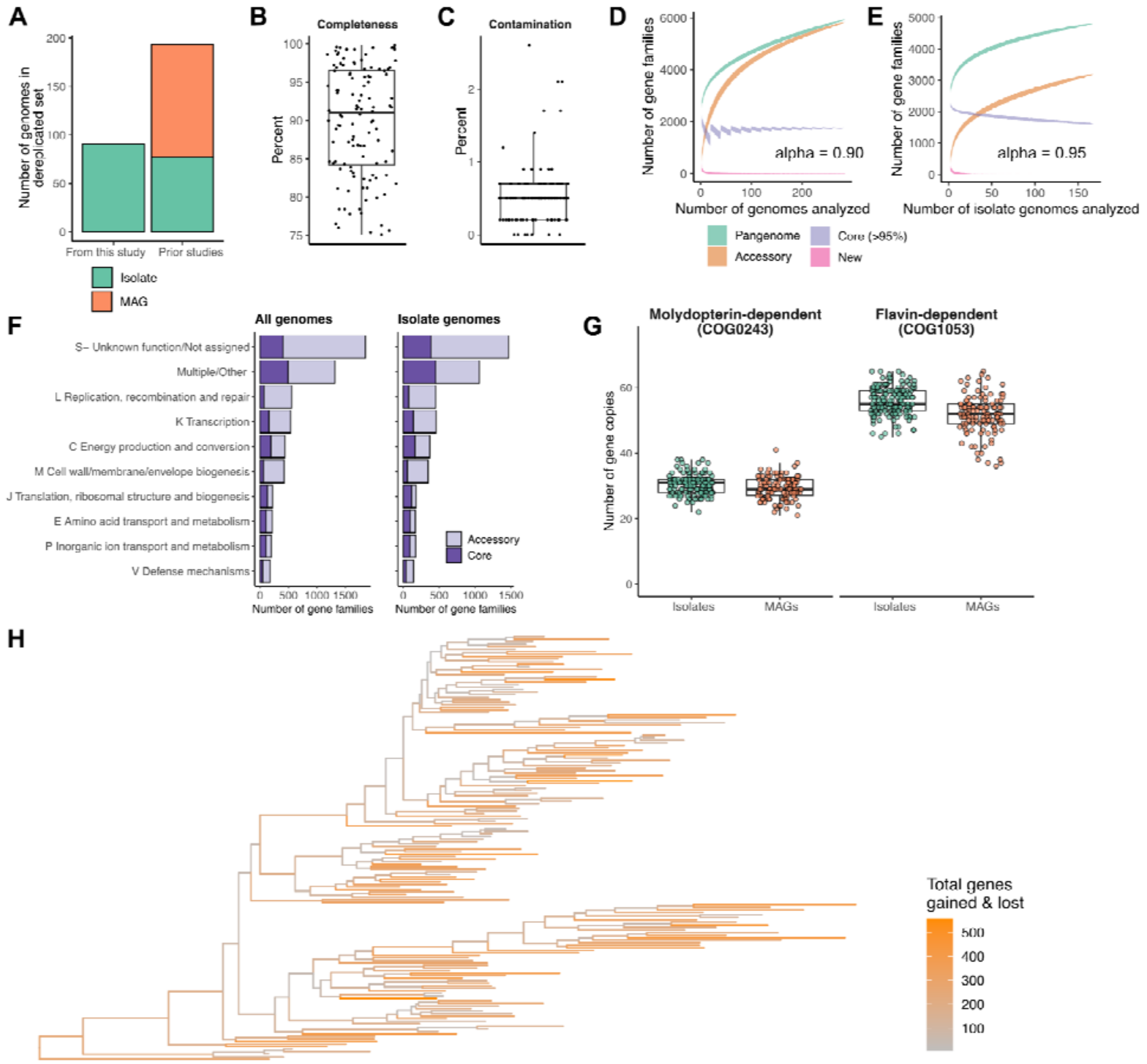

bioRxiv preprint doi: https://doi.org/10.64898/2026.03.31.715625; this version posted April 1, 2026. The copyright holder for this preprint (which was not certified by peer review) is the author/funder, who has granted bioRxiv a license to display the preprint in perpetuity. It is made available under aCC-BY 4.0 International license.

Figure S2. *E. lenta* pangenome variation. (A-C) Genomes analyzed in this study, including their sources **(A)** and for MAGs, their completeness **(B)** and contamination **(C)** statistics (BUSCO[79]). **(D,E)** Pangenome sampling curve showing the accumulation of total genes (green), accessory genes (orange), core genes (purple), and new genes (pink) as a function of genomes analyzed for **(D)** all genomes (95% cutoff for core genome inclusion) and **(E)** isolate genomes (100% prevalence cutoff). Band width represents the standard deviation across samplings (*n*=284 for all genomes and 168 for isolates only). Each curve is labeled with the alpha parameter estimated by a Heaps' law model with 500 iterations[88,89]. Values <1 indicate an open pangenome. **(F)** Distribution of core and accessory gene families (eggNOG) across the most common COG categories for all genomes (95% cutoff for core genome inclusion) and isolate genomes (100% prevalence cutoff). Category S (Unknown function) was combined with unassigned gene families. **(G)** Variation in copy number of genes from two anaerobic reductase superfamilies among isolate genomes and MAGs. **(H)** Maximum likelihood phylogenetic tree (**Figure 1A**), recolored by the estimated number of gene families gained and lost at each node of the tree (*Panstripe*[90] phylogenetic model).

bioRxiv preprint doi: https://doi.org/10.64898/2026.03.31.715625; this version posted April 1, 2026. The copyright holder for this preprint (which was not certified by peer review) is the author/funder, who has granted bioRxiv a license to display the preprint in perpetuity. It is made available under aCC-BY 4.0 International license.

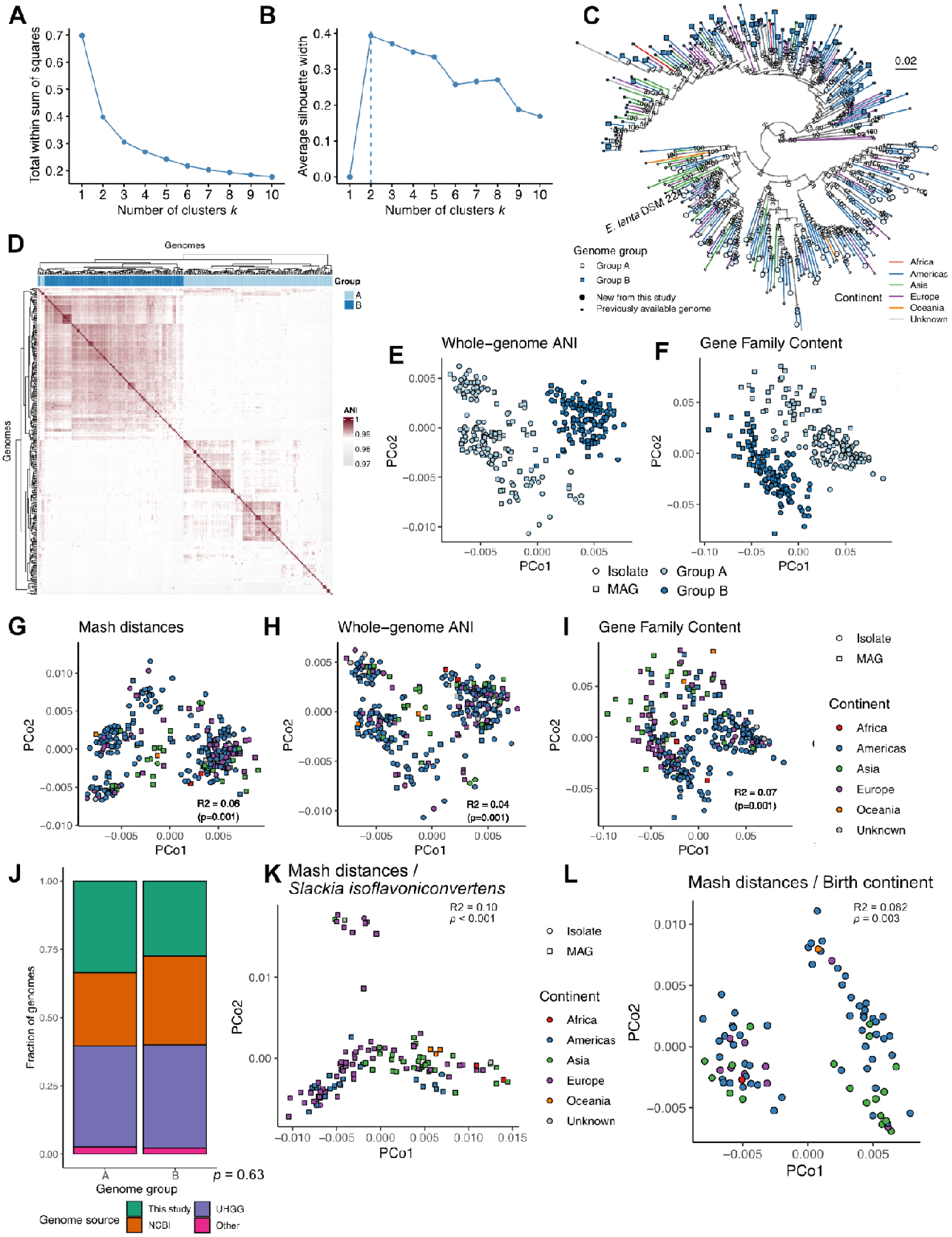

bioRxiv preprint doi: https://doi.org/10.64898/2026.03.31.715625; this version posted April 1, 2026. The copyright holder for this preprint (which was not certified by peer review) is the author/funder, who has granted bioRxiv a license to display the preprint in perpetuity. It is made available under aCC-BY 4.0 International license.

Figure S3. *E. lenta* genomes cluster into two groups with a limited phylogeographic signal. (A-B) Elbow (**A**) and silhouette (**B**) plots evaluating the optimal number of genome clusters in our dataset, based on Mash sequence dissimilarity. **(C)** Maximum likelihood phylogenetic tree of the *E. lenta* species based on a core gene alignment generated by *Panaroo*[95]. Bootstrap values were estimated for each node using RAxML. Tips shown in a larger size represent new genomes from this study, while tip shape indicates genome group. Branch colors indicate geographic origin. **(D)** Hierarchical clustering of genomes based on whole-genome average nucleotide identity (ANI), showing a division into two main groups. **(E-F)** Principal coordinates analysis of genomes based on whole-genome ANI (**E**) and eggNOG gene family presence-absence (**F**), illustrating division into groups A and B. **(G-I)** Principal coordinates analysis based on sequence Mash distances (**G**), whole genome ANI (**H**), and eggNOG gene family presence-absence (**I**), as in **Figure 1B** and **Figure S3E-F**, but colored by geographic origin. Effect sizes and *p*-values are shown for the association between dissimilarity and continent of origin. **(J)** Bar graph showing the fraction of genomes in groups A and B from each of the major sources for this study. *p*-value based on Fisher's exact test. **(K)** Principal coordinates analysis based on Mash distances of genomes from a related *Eggerthellaceae* species, *Slackia isoflavoniconvertens*. **(L)** Principal coordinate analysis of *E. lenta* genomes from the ImmunoMicrobiome cohort[31] based on sequence Mash distances, colored by host continent of birth. Effect size and *p*-values in **E-I, K,L** are based on PERMANOVA. Color and shape legends are shared between panels **K** and **L.**

bioRxiv preprint doi: https://doi.org/10.64898/2026.03.31.715625; this version posted April 1, 2026. The copyright holder for this preprint (which was not certified by peer review) is the author/funder, who has granted bioRxiv a license to display the preprint in perpetuity. It is made available under aCC-BY 4.0 International license.

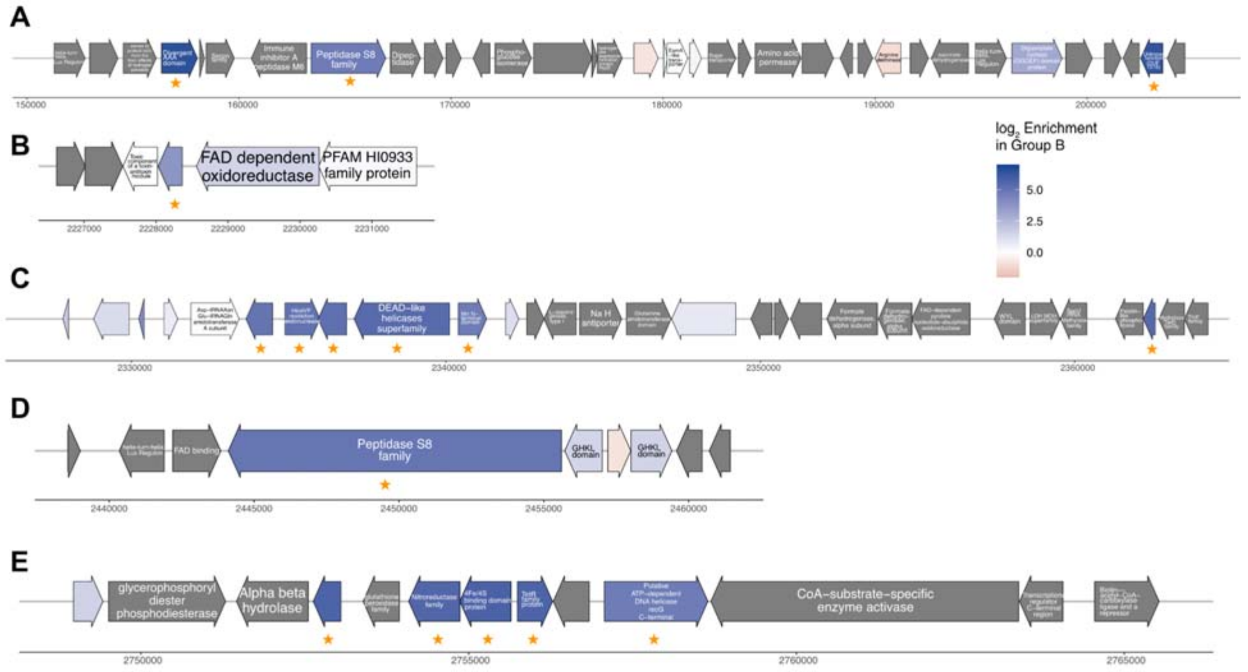

Figure S4. Identification of multiple genomic loci containing Group B enriched genes. (A-E) Genomic context for the 16 gene families strongly linked to *E. lenta* Group B (**Table S3, Methods**). Gene maps are based on *E. lenta* APC055-928 and ordered based on coordinates in this genome. Gold stars indicate top Group B-linked genes. Genes are labeled with eggNOG gene family descriptions and colored based on their enrichment in Group B vs. Group A. Genes shown in gray are part of the core genome.

bioRxiv preprint doi: https://doi.org/10.64898/2026.03.31.715625; this version posted April 1, 2026. The copyright holder for this preprint (which was not certified by peer review) is the author/funder, who has granted bioRxiv a license to display the preprint in perpetuity. It is made available under aCC-BY 4.0 International license.

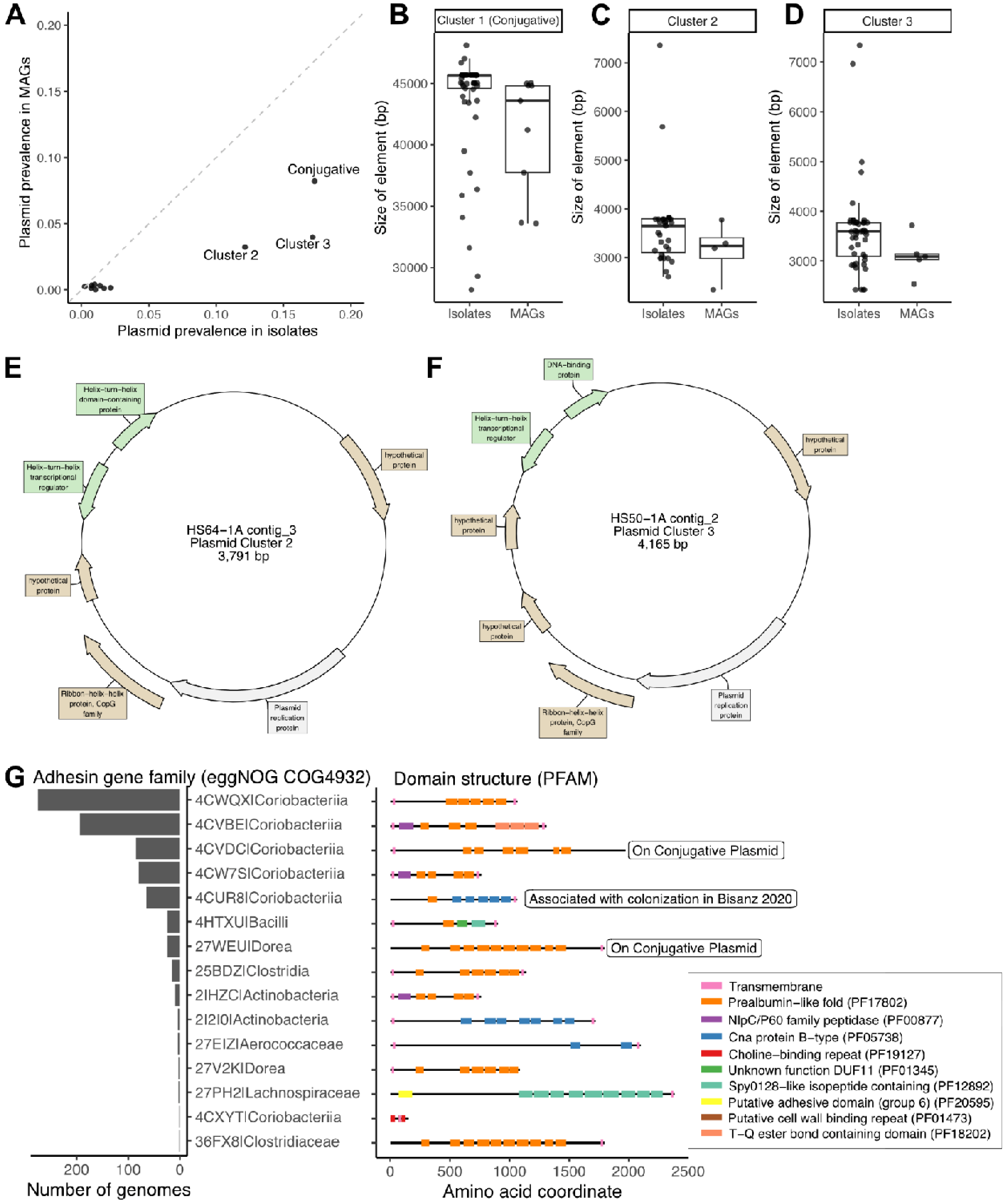

bioRxiv preprint doi: https://doi.org/10.64898/2026.03.31.715625; this version posted April 1, 2026. The copyright holder for this preprint (which was not certified by peer review) is the author/funder, who has granted bioRxiv a license to display the preprint in perpetuity. It is made available under aCC-BY 4.0 International license.

Figure S5. Discovery of three types of plasmids that are widespread in the *E. lenta* species. (A) Prevalence of plasmid clusters (sets of closely related plasmids found across genomes, **Methods, Table S5**) across both isolates and MAGs. The three clusters detected in at least 10 genomes are labeled. All clusters had higher prevalence in isolate genomes than in MAGs (below the dashed diagonal line indicating equal prevalence), as expected. **(B-D)** Plasmid element sizes are similar between MAGs and isolates. Boxplots show the distribution of contig sizes among elements in the 3 most prevalent clusters. **(E-F)** Representative gene maps showing gene annotations for the most common small plasmid clusters. Genes are colored by functional category (green = transcription, gray = replication, brown = unknown function). **(G)** Summary of annotated host-binding adhesin gene families found in the *E. lenta* pan-genome (belonging to COG superfamily COG4932). Gene families are labeled with the eggNOG subfamily ID and the associated eggNOG taxonomic assignment. For each gene family, the left panel shows their prevalence in the genome collection, while the right panel shows their PFAM domain structure as annotated by InterProScan (right panel). Two gene families that are only found on the conjugative plasmid are labeled, as is a related gene family that was associated with impaired fitness within the gastrointestinal tract[29].

bioRxiv preprint doi: https://doi.org/10.64898/2026.03.31.715625; this version posted April 1, 2026. The copyright holder for this preprint (which was not certified by peer review) is the author/funder, who has granted bioRxiv a license to display the preprint in perpetuity. It is made available under aCC-BY 4.0 International license.

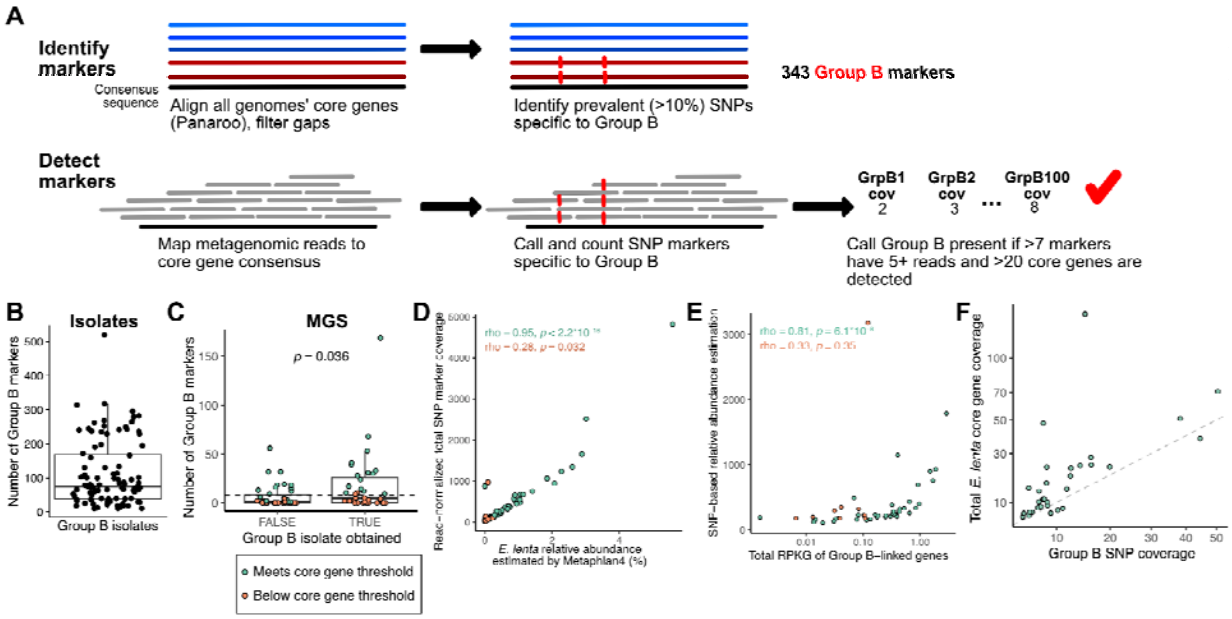

Figure S6. Use of core gene alignment to distinguish *E. lenta* Group B in metagenomic datasets. (A) Method used to quantify *E. lenta* Group B. Core genes were retrieved from isolate genomes and aligned to identify SNP markers found in >10% of Group B genomes and absent in the rest of the *E. lenta* species. Metagenomic reads were then aligned to the core gene consensus and the presence of Group B-specific SNP markers was used to determine its presence. **(B,C)** The number of distinct Group B SNP markers varies between isolate genomes in this group **(B)** and is higher in metagenomic data from samples where a Group B *E. lenta* isolate was obtained **(C)**. **(D)** Coverage of all SNP markers is correlated with Metaphlan-based *E. lenta* abundance (Spearman correlation). **(E)** Normalized SNP-based relative abundance of Group B (average coverage of core gene SNP sites per billion reads) is correlated with HUMAnN-based abundance of Group B-linked genes (reads per kilobase per genome equivalent, RPKG). **(C-E)** Colors indicate samples above (green) or below (orange) our minimum detection threshold (>20 core *E. lenta* genes). **(F)** Coverage of Group B SNPs is often lower than total coverage of *E. lenta* core genes. Each point shown represents a sample that met coverage thresholds for Group B detection. The dotted line is $y=x$. Both axes are on a square root scale to visualize low-abundance points.

bioRxiv preprint doi: https://doi.org/10.64898/2026.03.31.715625; this version posted April 1, 2026. The copyright holder for this preprint (which was not certified by peer review) is the author/funder, who has granted bioRxiv a license to display the preprint in perpetuity. It is made available under aCC-BY 4.0 International license.

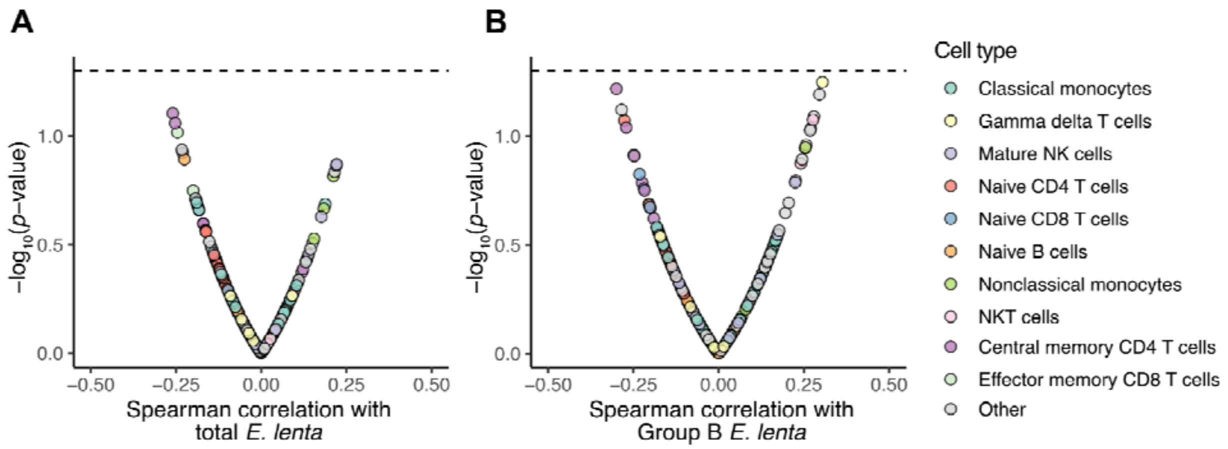

Figure S7. *E. lenta* is not associated with circulating immune cell populations in the ImmunoMicrobiome cohort. Volcano plots showing association of CyTOF cell population scaled cluster frequencies from serum samples, as described[31], with the SNP-based abundance (average coverage per billion reads) of the *E. lenta* species **(A)** or *E. lenta* Group B (**B**), based on core gene markers. Dotted line represents an uncorrected *p*-value of 0.05.
